# The OPTIMISE project: protocol for a mixed methods, pragmatic, quasi-experimental trial to improve primary care delivery to refugees in Australia

**DOI:** 10.1186/s12913-019-4235-6

**Published:** 2019-06-19

**Authors:** Grant Russell, Nilakshi Gunatillaka, Virginia Lewis, I-Hao Cheng, Joanne Enticott, Geraldine Marsh, Shiva Vasi, Jenny Advocat, Hyun Song, Shoko Saito, Sue Casey, Mitchell Smith, Mark Harris

**Affiliations:** 1 Department of General Practice, School of Primary & Allied Health Care, Faculty of Medicine, Nursing and Health Sciences, Monash University, Building 1, 270 Ferntree Gully Rd, Notting Hill, Victoria 3168 Australia; 2 Australian Institute for Primary Care and Ageing, College of Science, Health and Engineering, La Trobe University, Melbourne, Victoria Australia; 3 Enliven, Melbourne, Victoria Australia; 4 Centre for Primary Health Care and Equity, Faculty of Medicine, UNSW, Sydney, New South Wales Australia; 5 Foundation House, Melbourne, Victoria Australia; 6 New South Wales Refugee Health Service, Sydney, New South Wales Australia

**Keywords:** Migrant health, Refugee health, Primary health care, Primary care, Partnerships, Intervention trial

## Abstract

**Background:**

Australia is one of many nations struggling with the challenges of delivering quality primary health care (PHC) to increasing numbers of refugees. The OPTIMISE project represents a collaboration between 12 organisations to generate a model of integrated refugee PHC suitable for uptake throughout Australia. This paper describes the methodology of one component; an outreach practice facilitation intervention, directed towards improving the quality of PHC received by refugees in Australian general practices.

**Methods:**

Our mixed methods study will use a cluster stepped wedge randomised controlled trial design set in 3 urban regions of high refugee resettlement in Australia.

The intervention was build upon regional partnerships of policy advisors, clinicians, academics and health service managers. Following a regional needs assessment, the partnerships reached consensus on four core areas for intervention in general practice (GP): recording of refugee status; using interpreters; conducting comprehensive health assessments; and referring to refugee specialised services.

Refugee health staff trained in outreach practice facilitation techniques will work with GP clinics to modify practice routines relating to the four core areas.

36 general practice clinics with no prior involvement in a refugee health focused practice facilitation will be randomly allocated into early and late intervention groups.

The primary outcome will be changes in number of claims for Medical Benefit Service reimbursed comprehensive health assessments among patients identified as being from a refugee background. Changes in practice performance for this and 3 secondary outcomes will be evaluated using multilevel mixed effects models.

Baseline data collection will comprise (i) pre-intervention provider survey; (ii) two surveys documenting each practices’ structure and approaches to delivery of care to refugees. De-identified medical record data will be collected at baseline, at the end of the intervention and 6 and 12 months following completion.

**Discussion:**

OPTIMISE will test whether a regionally oriented practice facilitation initiative can improve the quality of PHC delivered to refugees. Findings have the potential to influence policy and practice in broader primary care settings.

**Trial registration:**

Australian New Zealand Clinical Trials Registry, ACTRN12618001970235, 05/12/2018, Retrospectively registered.

Protocol Version 1, 21/08/2017.

**Electronic supplementary material:**

The online version of this article (10.1186/s12913-019-4235-6) contains supplementary material, which is available to authorized users.

## Background

Australia is a major site for the resettlement of refugees. Over 78,000 permanent resident visas were granted to refugees and asylum seekers between 2011 and 2016 [1, 2]. The nation’s refugee and humanitarian program intake has recently increased (in 2018/19) to 18,750 per annum [2], the majority of whom will settle in the states of New South Wales and Victoria [3, 4].

The vulnerability of Australians of a refugee background follows from the physical and psychological sequelae of torture, trauma and multiple deprivations in countries of origin and transit [5]. Compared to the wider population, refugees are at greater risk of mental health conditions [6], infectious disease [7], nutritional deficiencies [8], obstetric complications [8], poor dental health [9] and disability [10]. Complex physical and psychological problems are often addressed only for the first time in Australia [11].

Australian states and territories have introduced different approaches to aid transition to life in a new country [3, 4]. Pathways vary across regions within different states/territories, and, as to whether refugees arrive through a sponsored or non-sponsored pathway. However, in general, resettled refugees are initially put in contact with Commonwealth funded Humanitarian Settlement Program service providers, which provide practical assistance for settling in the community. In many jurisdictions, new arrivals may be directed to Refugee Focused Health Services and/or a mainstream General Practice for initial health assessment [12]. Subsequently, all but the most complex clients are managed within mainstream primary care.

The health and wellbeing of people of refugee background is intimately linked with their ability to access high quality, coordinated primary health care [12]. Many struggle to access general practitioners, specialists, community health services and hospitals – problems related to socio-economic factors, language and cultural differences and the complexity of the Australian healthcare system [3, 4]. Provision of quality care to refugee background patients in mainstream primary care has been found to be inconsistent, with many providers having incomplete knowledge of refugee health requirements. Interpreters are underutilised, [5], coordination between sectors problematic, and, at times, in some jurisdictions, Refugee Focused Health Services have found it difficult to provide timely care [13, 14].

Outreach practice facilitation has become a promising intervention to support quality improvement in primary care [15]. Practice facilitation has been shown to improve chronic disease management, increase prevention, and facilitate system-level change and quality improvement [16]. Emerging evidence has shown that the principles of outreach practice facilitation could assist in the care of vulnerable populations, including refugees [17, 18]. This protocol outlines a project designed to investigate the effectiveness of outreach practice facilitation in supporting primary care practice-wide quality improvement within the context of refugee health.

### Objectives

Our overall objective is to conduct a mixed methods evaluation of a practice facilitation intervention designed to improve the primary care management of refugees in 36 general practice clinics across 3 regions: South East Melbourne (SEM), North West Melbourne (NWM) and South West Sydney (SWS).

We asked whether a facilitation-based intervention directed at improving the quality of general practice-based primary care delivered to patients with a refugee background could increase:

1. The conduct and documentation of comprehensive physical and mental health assessments among patients identified as being from a refugee background (primary outcome)

Secondary outcomes:

2. Refugee status determination and recording
3. The use of credentialed interpreters among patients identified as being from a refugee-like background
4. Practice staff knowledge and use of referral pathways to refugee specific health and social welfare services

## Methods

### Study design

This quality improvement programme has a mixed methods approach incorporating a cluster randomised controlled trial (cRCT) design with blind allocation to early and late intervention groups [19]. Quantitative measures collected cross-sectionally at multiple timepoints will be assessed using the recommended statistical approach for step-wedged cRCT designs [20]. An embedded qualitative component will explore the acceptability, usefulness and sustainability of the intervention(s).

The study will be informed by the literature on organisational change that identifies the factors influencing the diffusion and institutionalisation of organisational innovations, and where organisations are considered as a whole rather than as a set of independent attributes [21–23]. Two principles underpin the study design: a) Participatory research: where community based research partnerships built upon on-going knowledge exchange are considered an essential component of sustainable innovations and, community impact [24, 25]; and b) Implementation science: incorporating contemporary approaches to quality improvement, and, in particular Normalization Process Theory’s principles for understanding the process of embedding change in a practice by examining what people actually do and how they work [26].

### Study setting

The study will be conducted in general practice clinics in two regions of metropolitan Melbourne and one region in metropolitan Sydney, each with high rates of refugee resettlement. The study regions correspond to the Commonwealth Government Primary Health Networks (PHN) regions of South East Melbourne, North West Melbourne and South West Sydney [27].

### Sample

Trial eligibility is at three levels. Participating general practice clinics will be located in one of the three PHN regions, provide general primary care services and plan to be in operation for the next two years without substantial change to governance or management. Each will have needed to be in operation for 12 months and use electronic medical records and billing software compatible with the study’s data extraction tool (PENCS CAT4**™**). Practices will not have participated in refugee health related capacity building activities in the 12 months immediately preceding the study.

At least 50% of GPs in a practice must consent for practice participation to be confirmed. As the intervention is designed around a whole-of-practice approach, the research team will ensure there is a process by which staff within the practice can confidentially object to the practice’s participation in the study, via call or email. If this occurs, the practice will be withdrawn from the study.

### Intervention

#### Development of the intervention

The oversight of the study’s activities in each region is the responsibility of regional partnership teams (RPTs) comprising decision makers, clinicians, academics, health service managers and community members (Table 1).

**Table 1:** Participating organisations

Regional Partnership	Academic Institutions	Primary Health Organisations	Refugee Focused Health Services	Settlement agencies	State based organisations	National organisations
South East Melbourne	Monash University	Enliven Victoria 2016–2017: South Eastern Health Providers Association	Monash Health Refugee Health and Wellbeing	AMES Australia	Victorian Department of Health and Human Services Victorian Refugee Health Network	Royal Australian College of General Practitioners Refugee Health Network of Australia
North West Melbourne	La Trobe University	North Western Melbourne PHN.	cohealth			
South West Sydney	University of New South Wales	South Western Sydney PHN.	NSW Refugee Health Service	Settlement Services International	NSW Refugee Health Service	

Each RPT worked to characterise each region’s policy, program and service context with particular reference to the care of refugee populations. We focused specifically on barriers and enablers affecting access, transition and quality of primary care within the region. To further understand regional partnership needs, each RPT performed a focused scientific and grey literature review, analysed secondary data extracted from the Australian Bureau of Statistics Census, Department of Home Affairs Settlement Reporting Facility, settlement agencies, public health services and local councils, and conducted a series of key informant interviews with health service managers, policy advisors and clinicians. The three RPTs then met in a deliberative forum [28] to determine priority interventions, from which four core areas of activity were chosen;

- Physical and mental health assessments: GPs and practice nurses will have relevant skills and knowledge to conduct comprehensive refugee physical and mental health assessments.
- Refugee identification: general practice clinics will have accurate and accessible mechanisms in place to record and retrieve refugee identification, status, country of birth or ethnicity, year of arrival, and need for interpreter.
- Use of interpreters: practices will have a practice protocol for using interpreters, and ensure that staff have appropriate knowledge and skills in their role of supporting use of interpreters**.**
- Referral pathways: practice staff will gain an understanding of refugee health referral pathways in the region (including available services, eligibility requirements and referral procedures).

These areas of focus helped the study objectives (see above). All practices recruited will be asked to work on these four core areas as a minimum, with the option to choose an additional 1–2 activities from a list of optional tasks depending on interest and capacity (Table 2).

**Table 2:** Intervention Optional activities

Activity	Description
Cultural awareness	Practice staff demonstrate cultural awareness and sensitivity to refugee issues, including an understanding of the refugee experience.
Communication skills	GPs and practice nurses have appropriate communication skills (They are sensitive to the needs of refugees and take time to explain care to patients so they can make informed decisions by understanding what is happening as part of their care).
Business practices	practice staff are knowledgeable about and use business practices (including longer appointments, booking appointment with specific GP, Medicare billing, etc.) to support conduct of refugee health assessments
Information sharing	Practice has in place clear processes for sharing relevant patient information with other services. Practice staff use these processes consistently when receiving patient information and obtaining patient information.
Follow up on referrals	Practice staff refer clients to appropriate services and check whether the client attended the service. (If the problem is urgent or clinically significant this follow up may be with the receiving service, otherwise, follow-up will occur when patient re-attends the clinic)
Clinical matters	Practices may also identify other areas related to the clinical care of refugees. GPs and practice nurses may choose to learn more about the diagnosis and management of specific refugee health issues, e.g. refugee catch-up immunisation, mental health, paediatric health, infectious diseases.

#### Intervention process

Practice Facilitators will deliver the intervention to general practice clinics using a plan/do/study/act (PDSA) approach oriented to the four core goals [29]. Practice Facilitators will make a minimum of three, 60–90 min, in-person contacts with each participating practice during the six-month intervention period. Practices will receive one telephone contact following each visit.

Prior to the intervention commencing, each practice will identify a team comprising the practice lead and key practice participants, including at least one GP and (if applicable) one practice nurse. The team will complete a survey designed to ascertain current practice performance and needs in key areas of refugee health. This information will be used to help the Practice Facilitator lead a discussion during their first visit to the practice where the practice team will identify and discuss potential needs and areas for action, and develop an action plan (following a pre-designed template) for each of the four core priority areas. The action plans were adapted from the Victorian Refugee Health Network’s *Engaging and supporting General Practice in refugee health* program [18] and an example can be found in Additional file 1.

The subsequent two practice facilitation visits and phone calls will act to support and monitor action plan development and implementation. The Practice Facilitator will continue to assist with challenges faced by the practice and identify any other resources, strategies, tools or training available to aid in the action plan’s implementation.

Practices will also be provided with additional resources relating to the four core priority areas, including a soft and hard copy of a “General Practice Resource Book”. This book was created by the research team to provide a directory of verified resources organised to complement the four goals targeted by the project. A two-page electronic ‘summary sheet’ of key resources, with links embedded to be easily accessed on electronic devices, will also be provided.

#### Intervention providers

Practice Facilitators will be existing refugee health staff in each region and will be refugee health clinical fellows,^1^ refugee health nurses^2^ or project officers currently employed by Refugee Focused Health Services. Each will need to have experience in refugee and asylum seeker health care delivery and an understanding of the principles of engaging general practice clinics in quality improvement activities. This knowledge as well as the tasks and timelines of the role will be reinforced by a one-day training program and on-going facilitator support meetings. The content of the training was based on findings from the North-West Melbourne Primary Health Network’s in-house GP support program, the Settlement Coordinators’ program, and the Victorian Refugee Health Network’s *Engaging and supporting General Practice in refugee health* program. Additional material was sourced from the Agency for Healthcare Research and Quality training program for outreach practice facilitation [30] and the researchers’ prior work in other facilitation initiatives [31–33].

### Outcomes

The primary outcomes will be changes in number of claims for the delivery of Medicare Benefits Schedule (MBS) reimbursed comprehensive health assessments among patients identified as being from a refugee background. We will calculate the change in proportion of eligible (within 12 months of arrival in Australia) refugee background patients with medical records where there is evidence of billing for a comprehensive health assessment (MBS items 701, 703, 705 and 707). Secondary outcome measures will be:

(i) The change in the proportion of patients with records with recorded refugee identification (country of birth, ethnicity, year of arrival).
(ii) The change in proportion of refugee patient consultations with documented use of credentialed interpreter, where one was required.
(iii) The change in practice staff knowledge, attitudes and behaviours towards referring refugee background patients to external health and welfare services for which their refugee patients are eligible.

We will also measure changes in general practitioners’ confidence to undertake health assessments, catch-up immunisation and infectious diseases screening, and changes in practice routines relating to health assessments.

### Participant timeline

A timeline of the intervention proposal is shown in Table 3.

**Table 3:** Intervention Timeline

Month	Pre-intervention		1st month Facilitation	2nd Facilitation	3rd Facilitation	4th Facilitation	5th Facilitation	6th Facilitation	7th mth	8th	9th	10th	11th	12th m	13th	
	July – August & ongoing as required	August ongoing	Grp 1 September +	Oct +	November +	December +	January +	Feb +	March +	April	May	Jun	July	August	Sept 2018 +	
			Grp 2 March 2018 +	Apr +	May +	Jun +	July +	Aug +	Sept +	Oct	Nov	Dec	Jan	Feb	March 2019 +	
Activity	Recruitment of 12 practices	Baseline data collection (with all practices following consent)	Practice facilitation visit #1 (Doesn’t commence until consent confirmed, & practice survey completed)	Follow up and phone call #1	Practice facilitation visit #2	Follow up phone call #2	Practice facilitation visit #3	Follow up phone call #3	Post intervention data collection			Practices conduct business as usual				6-month post intervention data collection
Description	Includes all contact up to the point of gaining informed consent from the practice and staff (GPs, nurses and others). Open and targeted invitations. Informed by RHN, RHFs, others. EOI form completed & eligibility checked.	Randomise practices to immediate start or wait. Visit ALL practices as they are recruited to finalise consent and confirm data collection processes, including PEN CAT and TIS. Make appointments for PENCAT data collection Sign consent form for TIS data Commencing Practices Explain to practice manager that on line surveys must be completed before facilitation can commence: -Practice survey -Clinicians: at least 50% of GPs. WAITING practices (6 month wait): RO Also Completes Refugee healthcare survey with practice team. Facilitator not present.	Step 1. Pre intervention refugee healthcare (RHC) survey conducted by RO with practice team to identify potential areas for action Facilitator observes and takes notes to assist them with step 2. Step 2. Facilitator leads discussion with practice team informed by the RHC interview and practice description survey. Purpose is to a) identify and discuss action areas aligned to intervention priorities, b) to commence action plan development WAITING practices (6 month wait): Refugee healthcare survey is REPEATED with practice team and step 2 undertaken when they commence facilitation.	Prior to phone call send practice draft action plans (based upon learnings from refugee health survey) The practice reviews action plans, edit if required and return to facilitator. Practice implements action plans. Phone Call- Review progress with action plan development and early challenges with action plan implementation.	Provide support for action plan development and implementation and monitor implementation	Provide support for action plan implementation and monitor implementation Document changes to the action plan that have occurred.	Provide support for action plan implementation and monitor implementation Document changes to the action plan that have occurred.	Provide support for action plan implementation and monitor implementation Document changes to the action plan that have occurred.	PENCAT data extraction Post intervention RHC survey			New practices & procedures operate				PENCAT data extraction Post intervention RHC survey/ interview

### Sample size

Given that our primary outcome measure is a proportion i.e. the proportion of refugee patients’ records that show a refugee health assessment was billed when it was indicated, then to detect a post-intervention increase in percentage by 25–30% (a medium-large effect) we need approximately 12 clinics per region. In each of the 12 clinics we require cross-sectional data from 30 refugee background patients at each timepoint. Sample size calculations were done using Stata statistical software stepped-wedge for clusters defined at the level of the practice, intra-class correlation coefficient of 0.05, 80% power, an alpha of 0.05 and data collected at two time points [34].

### Recruitment

The project will be promoted to general practice clinics through the partner organisations, including practice visits, newsletters and promotion through each region’s PHN website. Practices will be invited to express interest through contacting the research team. Practices will be recruited by personal contact from the study’s research officers during an on-site visit to practices that express interest. The visit will allow an opportunity to further explain the project and to obtain written informed consent. Once the practice gives informed consent, clinical staff will be approached directly (generally through a practice meeting) and asked to provide written informed consent for the practice to participate in the intervention.

Practices will be offered a $2000 honoraria in recognition of the time required by staff to participate in the study. Participating general practitioners, practice nurses and practice managers will be eligible for continuing professional development points through their respective peak and professional bodies.

### Assignment of interventions

#### Allocation

Following recruitment into the study, practices will undergo blinded, stratified random allocation into either an early or late intervention group using a minimization procedure. Early group practices will start receiving the intervention immediately after recruitment. Late group practices will start the intervention 6-months after the date of completing the initial baseline data collection to act as control for the group receiving the intervention first. To ensure that early and late study groups are similar, practices will be stratified based on region and practice size as determined by the number of full time equivalent (FTE) general practitioners (≤ 5 FTEs, > 5 FTEs). The randomization codes were generated using Minim randomization software [35]. Wherever possible, batches of two or more practices will randomised at the same time.

#### Blinding

A statistician external to the project will perform the randomisation but will be blinded to the name or location of the practice. Practices will be informed of their allocation as blinding of practices to early or late intervention will not be possible.

### Data collection methods

Evaluation of the impact of the intervention will be assessed at the level of the practice. The researchers will collect data from early intervention practices at three-time points: at baseline (prior to the start of the intervention), at 6-months (immediately after completion of the intervention), and at 12 months (6-months after the intervention has been completed). Data collection in the late intervention practices will follow the same schedule, however they will have an additional point of data collection immediately prior to the start of the intervention. Table 4 provides an overview of the data collection methods used at each time point.

**Table 4:** Data Collection Timeline

	Practice Description Survey	Refugee Health Survey	Provider Survey	PENCS CAT4™ extract
Pre-intervention (Baseline)	x^a^	x	x	x
Post-intervention	–	x	x	x
6-months post intervention	–	–	–	x

^a^Late intervention group had two sets of baseline data collected; once at the initial recruitment and the other just before intervention began

A qualitative study is embedded within the quasi-experimental randomised controlled trial. We will use qualitative methods to generate an in-depth understanding of practice facilitation as the mechanism of change, and the key contextual determinants of change. Qualitative data will illuminate our understanding of variations in quantitative practice performance data in relation to refugee identification, interpreter use, conduct of comprehensive health assessments and referral, in response to the intervention.

The following instruments will be used to collect data:

- Quantitative measures

- **De-identified data extraction**: The PENCS CAT4**™** tool, is widely used by practices and Primary Health Networks to monitor practice performance and inform routine quality improvement interventions. The research team designed an add-on software report within the CAT4**™** tool to allow measurement of general practice performance in key refugee health quality domains including refugee identification, interpreter use and health/mental health assessments. Searches of each participating practice’s electronic clinical and practice management software will first filter all patient records where identifiers of country of birth, ethnicity or language spoken that are relevant to refugee populations have been recorded in a coded field or as free text in the patient record. All identifiable information including patient name, address, contact details will be automatically removed prior to the output being made available to the researchers. This output will be a de-identified line listed excel spreadsheet that includes (where available) residential postcode, age, gender, country of birth, ethnicity, language spoken, year of arrival, interpreter needed, list of diagnoses, date of first visit, dates of visits in the last 12 months, dates of visits where an interpreter was used, date of last health assessment and date of last mental health care plan. This data extract will be carried out at baseline and repeated at the end of the intervention period as well as at 6-months post intervention completion. Analysis will allow us to identify track changes in practice performance as a result of the quality improvement intervention.
- A **Practice Description survey** (Additional file 2) will be administered to the practice team at baseline and will contain questions regarding a practice’s staffing, patient load and demographics, organisational structure/governance, appointment setting systems, clinical record management systems, payment systems and processes for client transition including transfer of information. Survey items were primarily derived from the Preventive Evidence into Practice study (PEP) [33] and the Canadian Community-Based Primary Health Care Common Indicator Project [36].
- **A Practitioner survey** (Additional file 3) will be administered to each consenting general practitioner. This will contain questions ascertaining practice staff’s background, experience and interest in refugee health care delivery, and experience of and attitude towards using interpreters in clinical care. Survey items were derived from the Comparison of Models of Primary Care in Ontario study [37]; PEP [33]; the Community-Based Primary Health Care Common Indicator Project [36]; and the Patient-Centered Medical Home Scale [38]. The survey will be repeated at the end of the intervention period.
- **A Refugee healthcare survey** (Additional file 4) will be administered to each practice and their staff participating in the project by the Research Officer during the first facilitation visit to document whole-of-practice approaches to refugee care. The survey items were designed specifically for this project, informed by the areas of focus emerging from the deliberative forum. The data generated by using this tool will also inform tailoring of the practice facilitation intervention. The survey will be repeated at the end of the intervention period.

b) Qualitative data

- **Semi-structured interviews**: Following the intervention period, we will interview Practice Facilitators (*n* = 3), research officers (n = 3) and practice staff who played a key role in intervention from two practices in each round within each region (*n* = 12 practices) to explore their experiences of being involved in the intervention. The aims of the interviews are to identify key factors affecting the fidelity, effectiveness and sustainability of the intervention.
- **Facilitator diary:** Practice Facilitators will maintain a reflective diary of their contacts with practices to document activities undertaken, resources provided, challenges encountered and how these were overcome.
- **Action plans***:* As part of the intervention, Practice Facilitators will work alongside key practice personnel to document practice goals and relevant activities relating to refugee identification, interpreter use, conduct of comprehensive health assessments and referral. Action plans will be used to monitor progress towards practice goals and (where applicable) the documentary evidence of achieving these goals.
- **Facilitator meeting minutes***:* The research team will maintain detailed minutes from the research officers’ meeting and the Practice Facilitator meetings.
- **Reports of regional needs assessments***:* Will provide additional information on the region, including refugee population demographics, distribution general practice clinics and refugee health services within the catchment and socio-political factors affecting service provision.

### Data management

Survey data will be collected using electronic or paper based forms. All survey data will be entered into Qualtrics then checked for accuracy and completeness prior to de-identification. Patient data will be extracted from practice records using PENCS CAT4 in de-identified format. Trained researchers will clean quantitative data using a protocol created in collaboration with the project biostatistician. Quantitative data will be exported to SPSS or Stata for analysis.

Audio files of interviews with practice staff will be de-identified and transcribed verbatim. Interview transcripts, facilitator diaries and other documents will be exported to NVivo for analysis.

### Statistical methods

Changes in practice performance relating to the primary outcome measure will be assessed between early and late intervention practice groups using multilevel mixed effects models to account for clustering as recommended for stepped-wedge cRCT designs [19, 20]. All quantitative outcome measures will be assessed for clustering, and if not significant may be further explored through uni-level multivariate methods (e.g. logistic regression to investigate factors affecting the proportion of consultations with interpreter use). However, if practice-level clustering is indicated, then the mixed models will include a cluster-specific random effect to deal with clustering at the practice level. In the main analysis the independent variables defined as fixed effects will be group allocation (1 = early, 0 = late), intervention status (0 = pre-intervention, 1 = post-intervention) and timepoint (0 = baseline for all practices, 1 = post intervention for early practices, pre-intervention for late start practices, 2 = 6 months post intervention for early practices, post intervention for late start practices, 3 = 6 months post intervention for late practices). Timepoint will be included as a categorical variable. In subsequent analyses, provider and practice level covariates thought to influence outcomes will be included to adjust for baseline differences between the two groups.

### Qualitative analysis

A Research Fellow with qualitative methods expertise will read de-identified interview transcripts, facilitator diaries, and other documents. We will begin by developing a preliminary coding template, based on the initial reading and familiarisation with the raw data, as well as a priori broad theoretical concepts from Stange and Glasgow’s Context Tool [39] and May’s Normalisation Process Theory [26, 40]. We will refine and expand on the preliminary coding template following the standard qualitative techniques of immersion, crystallisation and constant comparison which involves repeated cycles of detailed examination of data, followed by reflection, identification, and articulation of themes and concepts and purposive refinement of existing theories. Interpretive insights are recorded to generate a ‘thick’ description of the findings and their context and include extracts from raw data and discussed with the team. Individual member of the research team will independently code a number of sources to test for reliability and the appropriateness of the final coding template.

We will use matrices to help organize and analyse the data and generate case descriptions [41]. We will evaluate the impact of the practice facilitation approach by exploring mechanisms for changes in practice routines across the four foci of the intervention, and then further considered against:

- Practice outer context: refugee background population in the area, links with external environment.
- Practice inner context: practice size, practice staff, time required to recruit the practice, proportion of refugee patients, staff/patient language concordance.
- Intervention: practice’s level of engagement with the intervention.
- Facilitator and research officers: the time research officers spend engaging the practices and troubleshoot issues arising with implementation.

Reliability will be informed by parallel collection of the same categories of data by multiple researchers, inter-rater comparisons of data coding decisions and clearly defined measurement procedures. Accuracy of the findings is achieved through the use of multiple data sources, and the triangulation of qualitative data with surveys and de-identified aggregate clinical data [42].

### Data monitoring

Minimal risks were evaluated with this study and hence a data monitoring committee was not needed.

#### Harms

Harms within the study are not anticipated, however in the event that they do arise, practices will inform the research officer and the practice may have to be withdrawn from the study. Ethics procedures are in place such that if a participating provider becomes distressed at interview they will be referred for psychological support.

#### Auditing

No plans for auditing have been made for this study.

### Ethical considerations

#### Research ethics approval

The project was approved by the Monash University Human Research Ethics Committee (Ref:10086), Monash Health Human Research Ethics Committee (Ref: 17-487L), South West Sydney Local Hospital District Human Research Ethics Committee (Ref: LNR/17/Lpool/391) and La Trobe University Human Research Ethics Committee (Ref: S17–138). Original protocol approvals were granted in September 2017 before project commencement. All protocol amendments require agreement by all authors, and will be submitted to and approved by the respective Human Research Ethics Committee using the appropriate ethics amendment forms. As a practice-based intervention designed to achieve organisational change, there was no direct contact with refugee clients, hence ethical concerns that may arise about conducting research about this vulnerable group were not applicable.

#### Consent or assent

Written informed consent will be obtained from participants.

#### Confidentiality

Data will be stored on password protected secure drives on Monash University servers, and/or in secure filing cabinets for the duration of the study. Data will be cleaned and de-identified by each site prior to secure transfer to the biostatistician.

#### Access to data

Investigators are data custodians during and after the project. Custodians need to be informed about any data management/analyses.

#### Ancillary and post-trial care

As there are low risks of harm with this study, ancillary and post-trial care has not been organised.

### Dissemination policy

The results of this study will be disseminated via publication in a peer reviewed journal and presented at a relevant conference. The investigators have adopted an authorship protocol based on the International Committee of Medical Journal Editors recommendations [43]. Final decisions in case of disputes regarding authorship rest with the study’s principal investigator. Partner agencies will be acknowledged in all publications. Professional writers will not be used. Findings will be shared with each Regional Partnership Team, and summaries of key findings will be shared with participating practices and other interested stakeholders. Public access will be provided for individual participant data that underlie the results reported in study publications, after de-identification (text, tables, figures, and appendices). We will also make available the statistical analysis plan and relevant statistical code. Availability will begin 6 months after and end 36 months following article publication and will be for proposals that have been approved by an independent review committee. Qualitative data will not be made publicly available.

## Discussion

A co-designed, practice facilitation intervention is an innovative approach to improve key refugee background patient outcomes in 36 practices in regions with the highest refugee settlement in Australia.

The findings of the research described in this paper will generate evidence for the effectiveness and limitations of a practice facilitation approach to quality improvement in general practice. This will add to the evolving body of knowledge around the implementation of practice facilitation, particularly whole-of-practice changes that impact on access to quality primary health care for vulnerable populations. It will also generate evidence about the approach to facilitate improvements in the delivery of primary care to people of refugee background.

Data on the intervention will generate valuable knowledge concerning the effectiveness of outreach practice facilitation in optimising the primary care of people of refugee background in Australia. If successful, it will deliver quality, accessible and coordinated PHC care to refugees. Primary care clinicians, especially those working within general practice clinics will benefit from insights for improving refugee identification, health assessment, interpreter use and referral. Potential solutions can be adapted to local contexts to address a local health system’s priority gaps and will ultimately assist the Australian health system to integrate care and reduce the burden on refugee focused health services to meet the needs of this vulnerable population. Additional benefits are likely to flow from inter-sectoral partnerships between academics, Refugee Focused Health Services, mainstream general practice clinics, settlement services, state governments and primary health care organisations.

## Additional files

Additional file 1: Action plan template (PDF 122 kb)

Additional file 2: Practice Description Survey (PDF 246 kb)

Additional file 3: Individual Clinical Staff Survey (PDF 419 kb)

Additional file 4: Baseline Refugee Healthcare Survey (PDF 475 kb)

## Data Availability

Not applicable.

## Abbreviations

cRCT: cluster Randomised Controlled Trial
FTE: Full Time Equivalent
GP: General Practice
MBS: Medicare Benefits Schedule
NWM: North West Melbourne
PDSA: Plan/Do/Study/Act
PHC: Primary Health Care
PHN: Primary Health Network
RPT: Regional Partnership Team
SEM: South East Melbourne
SWS: South West Sydney

## Footnotes

1 Refugee health fellows are medical practitioners funded by the Victorian Department ofHealth and Human services to build capacity in refugee and asylum seeker health. Moreinformation on refugee health fellows can be found at: http://refugeehealthnetwork.org.au/engage/refugee-health-fellows/

2 Refugee health nurses in Victoria are located in community health centres in areas of high refugee settlement. In New South Wales, they are affiliated with the NSW Refugee Health Service. Both programs are funded by the state Departments of Health and Human Services.
