# Supplementary material for: The OPTIMISE project: protocol for a mixed methods, pragmatic, quasi-experimental trial to improve primary care delivery to refugees in Australia
Source: BMC Health Serv Res. 2019 Jun 19;19:396. doi: 10.1186/s12913-019-4235-6 (PMC6585095; doi:10.1186/s12913-019-4235-6)
Supplement: Supplementary file 1 — Action plan template (PDF 122 kb) [file 12913_2019_4235_MOESM1_ESM.pdf]

Action plan template

GOALS<br><br>Priority intervention from list above. E.g. A whole of practice protocol for using interpreters, and staff with appropriate knowledge and skills in their role supporting use of interpreters						
Practice characteristics: e.g. practice size/governance, refugee population characteristics, availability of bilingual staff that may affect the practice's choice of objectives and actions.						
**Objectives**	**Existing Practice resources and new resources**	**ACTIONS TO ACHIEVE GOAL**	**Completion TARGET DATE**	**WHO IS RESPONSIBLE**	**WHAT EVIDENCE CAN WE USE TO SHOW IF WE'VE BEEN SUCCESSFUL?**	**PROGRESS**
SMART objective that fits with priority intervention area above<br><br>e.g. Improve usage of interpreters by all staff in the practice	List of resources that practices may use to achieve objective. Resources will be reviewed for quality and contextual appropriateness prior to inclusion on this list.<br><br>e.g. TIS Doctor's Priority Line brochure	Task #1<br><br>e.g. Register all GPs in the practice with individual TIS client codes.	November 2017	e.g. Practice manager	All GPs are registered with individual TIS client codes	
		Task #2				
		Task #3				
