# Supplementary material for: The OPTIMISE project: protocol for a mixed methods, pragmatic, quasi-experimental trial to improve primary care delivery to refugees in Australia
Source: BMC Health Serv Res. 2019 Jun 19;19:396. doi: 10.1186/s12913-019-4235-6 (PMC6585095; doi:10.1186/s12913-019-4235-6)
Supplement: Supplementary file 2 — Practice Description Survey (PDF 246 kb) [file 12913_2019_4235_MOESM2_ESM.pdf]

Practice Description Survey

Version date: 11th September 2017

Thank you for taking the time to complete this survey. Your responses to this survey will help us to understand how your practice is currently organised, what information systems are used, who works here and what kinds of policies and procedures you have in place for working with patients. When responding to the questions, please feel free to involve others in the practice, including the OPTIMISE Practice Team members.

Q1 **Name** of practice

____

____

Q2 **Address** of practice

____

____

Q3 What is your **name and contact number** (We will get in touch with you if we have any questions of clarification.)

____

____

Q4 What is **your role** in the practice

Q5 Please indicate **who else in the practice helped to complete the survey** (if relevant)

SECTION A: About Your Practice Staff
These questions will help us to understand how OPTIMISE works in different contexts, including the size and staffing levels and profession mix of practices.

We are asking for the <u>names</u> of staff to help us to ensure consent has been documented.

Q6 Please list the name(s) of the <u>**general practitioners**</u> who work in your practice:

	Name of General Practitioner(s)	Gender	Languages spoken	Sessions or half-days per week
1				
2				
3				
4				
5				
6				
7				
8				
9				
10				

Q7 Does your practice employ <u>**practice nurses**</u>?

- ○ Yes. *Please go to Q7.1*
- ○ No. *Please go to Q8*

Q7.1 Please list the name(s) of the <u>**practice nurses**</u> who work in your practice:

	Name of Practice Nurse(s)	Gender	Languages spoken	Sessions or half-days per week
1				
2				
3				
4				
5				

Q8 Please list the name(s) of the **practice managers** who work in your practice: A practice manager is responsible for the business management of the practice

	Name of Practice Manager(s)	Gender	Languages spoken	Sessions or half-days per week
1				
2				
3				
4				
5				

Q9 Does your practice employ **reception or administrative staff**?

◯ Yes. *Please go to Q9.1*

◯ No. *Please go to Q10*

Q9.1 Please list the name(s) of the **receptionist and administrative staff** who work in your practice:

	Name of Receptionist or Administrative Staff Member(s)	Gender	Languages spoken	Sessions or half-days per week
1				
2				
3				
4				
5				

Q10 Are there any **other staff**, including allied health professionals, who work in your practice:

	Name	Discipline (e.g. physio, psychologist)	Gender	Languages spoken	Sessions or half-days per week
1					
2					
3					
4					
5					

SECTION B: Roles and functions performed by staff in the practice
These questions explore the way you allocate different tasks and functions in the practice. This will help to design and implement strategies/activities.

Q11 Do nursing staff have **access to medical records** within Medical Director or Best Practice?

◯ Yes, all nurses do

◯ No, no nurses do

◯ Some nurses do (please explain why your response in the box below)

____

Q12 **Appointments**

Please indicate **who is responsible** for the **following functions in the practice**, especially as they **relate to refugees**.

You can use the "Other" column and comments box below if you need to.

	Not done in our practice	Doctors	Practice nurse	Practice manager	Reception/admin	Other (please specify in comments box below)
1. Triage patients to decide the order of access to appointments based on urgency of need	☐	☐	☐	☐	☐	☐

Q12.1 Comments

____

____

Q13 **Consultation/support**
Please indicate **who is responsible** for the **following functions in the practice**, especially as they **relate to refugees**.
You can use the "Other" column and comments box below if you need to.

	Not done in our practice (1)	Doctors (2)	Practice nurse (3)	Practice manager (4)	Reception/ admin (5)	Other (please specify in comments box below) (6)
1.Establish whether a patient needs an interpreter	☐	☐	☐	☐	☐	☐
2.Book an interpreter	☐	☐	☐	☐	☐	☐
3. Provide or support direct clinical care with an interpreter present (in person or on ‘phone)	☐	☐	☐	☐	☐	☐
4. Co-ordinates the conduct of health assessments	☐	☐	☐	☐	☐	☐
5. Provide refugee health assessments	☐	☐	☐	☐	☐	☐
6. Provide immunisations	☐	☐	☐	☐	☐	☐

Q13.1 Comments

____

____

Q14 **Information systems**
Please indicate **who is responsible** for the **following functions in the practice**, especially as they **relate to refugees**.
You can use the "Other" column and comments box below if you need to.

	Not done in our practice	Doctors	Practice nurse	Practice manager	Reception/admin	Other (please specify in comments box below)
1. Maintain a refugee patient register	☐	☐	☐	☐	☐	☐
2. Collect and collate information about refugee relevant services	☐	☐	☐	☐	☐	☐
3. Maintain directories of available services & programs	☐	☐	☐	☐	☐	☐
4. Update clinical guidelines, pathways, protocols	☐	☐	☐	☐	☐	☐

Q14.1 Comments

____

____

Q15 **Follow up/Referral**
Please indicate **who is responsible** for the **following functions in the practice**, especially as they **relate to refugees**.
You can use the "Other" column and comments box below if you need to.

	Not done in our practice	Doctors	Practice nurse	Practice manager	Reception/admin	Other (please specify in comments box below)
1. Manage patient recall for abnormal test results	☐	☐	☐	☐	☐	☐
2.Follow up patients who do not attend a previously scheduled appointment in the practice	☐	☐	☐	☐	☐	☐
3. Arrange follow-up appointments at the practice	☐	☐	☐	☐	☐	☐
4. Follow up to confirm that patients attended referral to external service	☐	☐	☐	☐	☐	☐

Q15.1 Comments

Q16 **Staff development/quality improvement**
Please indicate **who is responsible** for the **following functions in the practice**, especially as they **relate to refugees**.
You can use the "Other" column and comments box below if you need to.

	Not done in our practice	Doctors	Practice nurse	Practice manager	Reception/admin	Other (please specify in comments box below)
1. Organise staff training	☐	☐	☐	☐	☐	☐
2. Audit medical records for quality improvement	☐	☐	☐	☐	☐	☐
3. Ensure patient education and other materials are available in languages other than English	☐	☐	☐	☐	☐	☐

Q26.1 Comments

____

____

Q17 Are there **other important functions** that are undertaken that are **relevant to providing care to people with refugee backgrounds** that are not mentioned above? If so, please describe here, and indicate whose role it is.

____

____

Q18 Use this space if you wish to **elaborate on any responses above**

SECTION C: About the systems in your practice

These questions help us to understand the context in which you will implement change.

Q19 What are the **opening hours** of the practice?

	Monday	Tuesday	Wednesday	Thursday	Friday	Saturday	Sunday
Hours							

Q20 Does the practice have an **appointment system**?

- ◯ Yes. *Please go to Q20.1*
- ◯ No. *Please go to Q21*

Q20.1 What is the booking interval (time allowed) for a **standard appointment** with the doctor?

- ◯ Less than 5 minutes
- ◯ 5-10 minutes
- ◯ 11-15 minutes
- ◯ 16-20 minutes
- ◯ More than 20 minutes

Q20.2 What is the usual booking interval (time allowed) for a **new patient appointment**?

- ◯ Less than 5 minutes
- ◯ 5-10 minutes
- ◯ 11-15 minutes
- ◯ 16-20 minutes
- ◯ More than 20 minutes

Q21 Do you **confirm appointments** with patients a before scheduled visits?

- ◯ Always or almost always. *Please go to Q21.1*
- ◯ Often. *Please go to Q21.1*
- ◯ Sometimes. *Please go to Q21.1*
- ◯ Rarely or never. *Please go to Q22*
- ◯ Don't know or not applicable. *Please go to Q22*

Q21.1 **How do you confirm appointments**? For example, by phone, email or SMS

____

____

____

Q22 Does the practice promote the **use of the patient held electronic health record** (that is, My Health Record) to patients?

- ◯ Yes. *Please go to Q22.1*
- ◯ No. *Please go to Q23*
- ◯ Don't know. *Please go to Q23*

Q22.1 How do you promote the patient held electronic health record to patients? e.g. who is responsible, what information is provided to patients?

____

____

Q23 Does your practice **use HealthPathways or Map of Medicine**?

- ◯ Always or almost always
- ◯ Often
- ◯ Sometimes
- ◯ Rarely or never
- ◯ Don't know

Q24 Does the practice offer patients **privacy** when requesting or providing personal information (to front-of-house/reception staff or clinicians)? For example taking the patient to a separate room when asking about their background, past medical care, etc. or provide sensitive information or advice.

- ◯ Yes
- ◯ No

Q25 Does the practice **bulk-bill** any patients?

- ◯ Yes. *Please go to Q25.1*
- ◯ No. *Please go to Q26*

Q25.1 Please describe **who is bulk billed** (e.g. health care card only, all with Medicare card)

Q26 How does the practice bill **refugee patients who do not have a Medicare card**?

Q27 Does the practice use the **Health Assessment Medicare items when providing care to refugee patients**? For example, the MBS Item Numbers 701, 703, 705, 707 (noting that these are not specific to refugees)

- ◯ Yes. *Please go to Q27.1*
- ◯ No
- ◯ Don't know

Q27.1 Please explain how you use these Health Assessment items.

Q28 Does the practice have **regular practice meetings for clinical staff**?

◯ Yes, please state how often ____

◯ No.

◯ Don't know

◯ Not applicable

Q29 Does the practice have **regular practice meetings for non-clinical staff**?

◯ Yes, please state how often and whether these are combined with clinical staff

____

◯ No

◯ Don't know

◯ Not applicable

Q28.1/29.1
If you responded 'NO' to Q28 or Q29, how do you communicate with staff about significant changes within the practice?

____

____

Q30 Does the practice use **professional translation & interpreting services**?

	Yes	No	Don't know	No need
By Telephone	◯	◯	◯	◯
Face-to-face	◯	◯	◯	◯

If you answered ‘YES’ to using professional translating and interpreting services via telephone or face-to-face, please go to Q30.1.
Otherwise, please go to Q31

Q30.1 Which **languages** do you most **commonly use**? Select up to 5 from the list below.

- [ ] Albanian
- [ ] Amharic
- [ ] Arabic
- [ ] Aramaic
- [ ] Assyrian or Assyrian Neo Aramaic
- [ ] Bari
- [ ] Bosnian
- [ ] Burmese
- [ ] Chaldean Neo Aramaic
- [ ] Chin and related languages
- [ ] Croatian
- [ ] Dan (Gio-Dan)
- [ ] Dari
- [ ] Dinka
- [ ] Farsi
- [ ] Hakka
- [ ] Hazaraghi
- [ ] Karen
- [ ] Karen S'Gaw
- [ ] Kirundi (Rundi)
- [ ] Krio
- [ ] Kurdish
- [ ] Nepali
- [ ] Nuer
- [ ] Oromo
- [ ] Pashto
- [ ] Persian
- [ ] Serbian
- [ ] Shilluk
- [ ] Somali
- [ ] Swahili
- [ ] Tamil
- [ ] Tigrinya
- [ ] Turkish
- [ ] Urdu
- [ ] Vietnamese
- [ ] Zophei

Q30.1.1 Please note **any other commonly used languages** here

____

Q30.2 Which **languages** are **most difficult to find professional interpreters** for? Select up to 5 from the list below.

- [ ] Albanian
- [ ] Amharic
- [ ] Arabic
- [ ] Aramaic
- [ ] Assyrian or Assyrian Neo Aramaic
- [ ] Bari
- [ ] Bosnian
- [ ] Burmese
- [ ] Chaldean Neo Aramaic
- [ ] Chin and related languages
- [ ] Croatian
- [ ] Dan (Gio-Dan)
- [ ] Dari
- [ ] Dinka
- [ ] Farsi
- [ ] Hakka
- [ ] Hazaraghi
- [ ] Karen
- [ ] Karen S'Gaw
- [ ] Kirundi (Rundi)
- [ ] Krio
- [ ] Kurdish
- [ ] Nepali
- [ ] Nuer
- [ ] Oromo
- [ ] Pashto
- [ ] Persian
- [ ] Serbian
- [ ] Shilluk
- [ ] Somali
- [ ] Swahili
- [ ] Tamil
- [ ] Tigrinya
- [ ] Turkish
- [ ] Urdu
- [ ] Vietnamese
- [ ] Zophei

Q30.2.1 Please note **any other languages** for which **finding professional interpreters is difficult** here

____

Q31 Availability of information in **appropriate languages**

	Yes - for all patients who need it	Yes - for some patients who need it	No	Don't know or not applicable
Does the practice provide information about clinic opening hours and services available in multiple languages?	○	○	○	○
Are waiting room signs written in languages other than English?	○	○	○	○

If you answered ‘YES – SOME PATIENTS’ please go to Q31.1
Otherwise, go to Section D

Q31.1 Please describe how you determine which patients need these services or strategies.

____

____

____

SECTION D: About the patients in your practice If you don't have the precise information, please "estimate" numbers and proportions to answer the questions in this section. You will be asked at the end of this section whether your estimates are based on your own views, discussion with others or checking the practice databases or records.

Practice Patient Profile

Q32 What is the (estimated) **total number of active patients** on the practice's books (that is, the number of patients visiting the practice approximately 3 times in the past 2 years)

____

Q33 What are the **predominant cultural or ethnic groups** in your practice, and what proportion of the practice would each group represent?

	Predominant cultural or ethnic group	Estimated % of all patients
1		
2		
3		
4		
5		

Q34 Can you estimate the proportion of **active patients** in the practice who have a r**efugee (or refugee-like) background?** (active = have visited 3 times in the past 2 years)

____

Q35 Please choose the **best description** of the **source of your information** from the following statements:

- ◯ The figures above are based on my own estimates
- ◯ The figures above are based on discussions with others in the practice
- ◯ The figures above are based on reviewing databases such as patient registers or auditing medical records

Q36 Use this space if you wish to elaborate on any responses above.

____

____

Patient Register and Recall

Q37 Does the practice **record the following information for new patients**?

	Always or almost	Often	Sometimes	Rarely or never	Don't know or not applicable
Employment status	◯	◯	◯	◯	◯
Healthcare card holder	◯	◯	◯	◯	◯
Country of birth	◯	◯	◯	◯	◯
Refugee or asylum seeker status	◯	◯	◯	◯	◯
Country of origin	◯	◯	◯	◯	◯
Year of arrival in Australia	◯	◯	◯	◯	◯
Need for an interpreter	◯	◯	◯	◯	◯
Preferred language	◯	◯	◯	◯	◯

Q38 **<u>Where in the medical record</u>** does the practice usually record the following information?

	Not routinely collected	Warning section of clinical record	Social history section of clinical record	Progress notes in clinical record	Defined fields in billing software	As free text in billing software	Other (please specify in comment box below)
Employment status	○	○	○	○	○	○	○
Healthcare card holder	○	○	○	○	○	○	○
Country of birth	○	○	○	○	○	○	○
Refugee background including asylum seekers	○	○	○	○	○	○	○
Country of origin	○	○	○	○	○	○	○
Year of arrival in Australia	○	○	○	○	○	○	○
Need for an interpreter	○	○	○	○	○	○	○

Q39 Comments

Q40 How does the practice know or confirm that a patient **needs an interpreter**?

- ☐ Patient is asked whether he/she needs an interpreter
- ☐ Patient requests an interpreter
- ☐ Other ________________________________

Q41 Do the following staff have **access to the information** the practice has recorded about **refugee or asylum seeker status**?

	Yes, usually	Yes, sometimes	No	Don't know or not applicable
Reception or administrative staff	○	○	○	○
Practice nurses	○	○	○	○
Doctors	○	○	○	○
Other, please specify	○	○	○	○

Q42 If you have any other information that you think it is important to capture, please include it here.

____

____

____

____

End of Practice Description Survey
