# Supplementary material for: The OPTIMISE project: protocol for a mixed methods, pragmatic, quasi-experimental trial to improve primary care delivery to refugees in Australia
Source: BMC Health Serv Res. 2019 Jun 19;19:396. doi: 10.1186/s12913-019-4235-6 (PMC6585095; doi:10.1186/s12913-019-4235-6)
Supplement: Supplementary file 3 — Individual Clinical Staff Survey (PDF 419 kb) [file 12913_2019_4235_MOESM3_ESM.pdf]

Individual Clinical Staff Survey

Version date: 11th September 2017

Baseline GP, Nurse and other clinical staff survey Thank you for taking the time to complete this brief survey. The information you provide in this survey will help the practice tailor the OPTIMISE intervention to meet the particular needs and strengths of your practice and staff.

SECTION A: ABOUT YOUR PATIENTS*Note: We are asking these questions about "your patients"—that is, the patients to whom you provide direct clinical care—because each GP or nurse in the practice may see a different mix of patients.*

Q1 How many **patients with a refugee background** have you provided care to over the past 12 months? (An estimate is fine.)

____

Q2 What are the most **common languages** spoken by your **refugee patients**? Select up to 5 from the list below.

- ☐ Albanian
- ☐ Amharic
- ☐ Arabic
- ☐ Aramaic
- ☐ Assyrian or Assyrian Neo Aramaic
- ☐ Bari
- ☐ Bosnian
- ☐ Burmese
- ☐ Chaldean Neo Aramaic
- ☐ Chin and related languages
- ☐ Croatian
- ☐ Dan (Gio-Dan)
- ☐ Dari
- ☐ Dinka
- ☐ Farsi
- ☐ Hakka
- ☐ Hazaraghi
- ☐ Karen
- ☐ Karen S'Gaw
- ☐ Kirundi (Rundi)
- ☐ Krio
- ☐ Kurdish
- ☐ Nepali
- ☐ Nuer
- ☐ Oromo
- ☐ Pashto
- ☐ Persian
- ☐ Serbian
- ☐ Shilluk
- ☐ Somali
- ☐ Swahili
- ☐ Tamil
- ☐ Tigrinya
- ☐ Turkish
- ☐ Urdu
- ☐ Vietnamese
- ☐ Zophei

Q2.1 Please note **any other commonly used languages** here

____

Q3 With what proportion of your refugee patients are **not fluent in English**? (Please provide your response as a % of your refugee patients)

________________________________%

Q4 With what proportion of your refugee patients who are not fluent in English do you use a formally trained interpreter (phone or in person)?
(Please provide your response as a % of your refugee patients)

_________________________________%

Q5 With what proportion of your refugee patients do you use **other means to interpret or translate** ? (Not including trained interpreters. Examples include bilingual staff; written or computer text; patient's family member or friend)
(Please provide your response as a % of your refugee patients)

__________________________________%

Q6 What **barriers**, if any, are there for your **use of interpreters**? Select all that apply.

- ☐ Cost
- ☐ Availability
- ☐ No systems in place
- ☐ Inability to get same sex interpreter
- ☐ Patient refusal
- ☐ Patient concern about confidentiality
- ☐ Issues with quality/training of interpreters
- ☐ Other, please specify

____

Q7 Do you use **HealthPathways or Map of Medicine for refugee care**?

- ◯ Always or almost always
- ◯ Often
- ◯ Sometimes
- ◯ Rarely or never
- ◯ Don't know

Q8 Do you use **online resources** such as those provided by the Victorian Refugee Health Network or NSW Refugee Health Service **for refugee care**?

- ◯ Yes. *Please go to Q8.1*
- ◯ No. *Please go to Section B.*
- ◯ Don't know. *Please go to Section B.*
- ◯ Not applicable. *Please go to Section B.*

Q8.1 Please give some **examples of online resources** that you use for **refugee care**

____

____

SECTION B: ASSESSMENT AND MANAGEMENT OF PATIENTS

Q9 In the past 12 months, how often did you have **difficulty finding other services or providers to refer your patients** with refugee backgrounds for the following (if applicable)?

	Always or almost always	Often	Sometimes	Rarely or never	Don't know	Not applicable
Social and settlement issues	○	○	○	○	○	○
Mental health problems including PTSD	○	○	○	○	○	○
Complex clinical problems	○	○	○	○	○	○
Infectious Diseases	○	○	○	○	○	○
Oral and dental health issues	○	○	○	○	○	○

Q10 If you have any comments to make about the question above, please use this space.

____

____

Q11 Please rate your **confidence to undertake the following activities** with your patients with refugee backgrounds.

	Not at all	Slightly confident	Somewhat confident	Moderately confident	Very confident	Not my responsibility
Undertake a Refugee Health Assessment	○	○	○	○	○	○
Assess requirements for catch-up immunisations	○	○	○	○	○	○
Screen or arrange for screening for infectious diseases (eg TB)	○	○	○	○	○	○
Arrange an interpreter (on phone or in person)	○	○	○	○	○	○

Q12 Please indicate the **extent to which you agree or disagree** with the following statements

	Strongly disagree	Disagree	Neither agree nor disagree	Agree	Strongly agree	No opinion or don't know
I enjoy treating patients with refugee backgrounds	○	○	○	○	○	○
I find it difficult to provide care to patients from a different cultural background to my own	○	○	○	○	○	○
I find it difficult to provide care to patients who speak a different language to my own	○	○	○	○	○	○
Using professional interpreter services interferes with delivery of healthcare	○	○	○	○	○	○

Q13 Please indicate the **extent to which you agree or disagree** with the following statements

	Strongly disagree	Disagree	Neither agree nor disagree	Agree	Strongly agree	No opinion or don’t know
People in this practice operate as a real team	○	○	○	○	○	○
When we experience a problem in the practice, we make a serious effort to figure out what's really going on	○	○	○	○	○	○
Leadership in this practice creates an environment where things can be accomplished	○	○	○	○	○	○

SECTION C: ABOUT YOU

Q14 What is your **name**?

____

Q15 What is your **role** in the practice?

- ◯ General practitioner
- ◯ Practice nurse
- ◯ Other, please specify ______________________________

Q16 How long have you worked in **a general practice setting**? Please provide your response as number of years to the nearest year

______________________Years

Q17 How **many sessions do you work per week** in a **general practice** setting?

	Number of sessions or 1/2 days per week (1)
In this general practice	
In other general practices (if applicable)	

Q18 What is your **ethnicity**?

____

Q19 What **languages other than English** do you speak?

Q20 In which **country were you born**?

◯ Afghanistan	◯ Botswana	◯ Democratic Republic of the Congo
◯ Albania	◯ Brazil	◯ Denmark
◯ Algeria	◯ Brunei Darussalam	◯ Djibouti
◯ Andorra	◯ Bulgaria	◯ Dominican Republic
◯ Angola	◯ Burkina Faso	◯ Dominica
◯ Anguilla	◯ Myanmar/Burma	◯ Ecuador
◯ Antigua & Barbuda	◯ Burundi	◯ Egypt
◯ Argentina	◯ Cambodia	◯ El Salvador
◯ Armenia	◯ Cameroon	◯ Equatorial Guinea
◯ Australia	◯ Canada	◯ Eritrea
◯ Austria	◯ Cape Verde	◯ Estonia
◯ Azerbaijan	◯ Cayman Islands	◯ Ethiopia
◯ Bahamas	◯ Central African Republic	◯ Fiji
◯ Bahrain	◯ Chad	◯ Finland
◯ Bangladesh	◯ Chile	◯ France
◯ Barbados	◯ China	◯ French Guiana
◯ Belarus	◯ Colombia	◯ Gabon
◯ Belgium	◯ Comoros	◯ Gambia
◯ Belize	◯ Congo	◯ Georgia
◯ Benin	◯ Costa Rica	◯ Germany
◯ Bermuda	◯ Croatia	◯ Ghana
◯ Bhutan	◯ Cuba	◯ Great Britain
◯ Bolivia	◯ Cyprus	◯ Greece
◯ Bosnia & Herzegovina	◯ Czech Republic	◯ Grenada

- Guadeloupe
- Guatemala
- Guinea
- Guinea-Bissau
- Guyana
- Haiti
- Honduras
- Hungary
- Iceland
- India
- Indonesia
- Iran
- Iraq
- Israel and the Occupied Territories
- Italy
- Ivory Coast (Cote d'Ivoire)
- Jamaica
- Japan
- Jordan
- Kazakhstan (92)
- Kenya (93)
- Kosovo (94)
- Kuwait (95)
- Kyrgyz Republic (Kyrgyzstan)
- Laos
- Latvia
- Lebanon
- Lesotho
- Liberia
- Libya
- Liechtenstein
- Lithuania
- Luxembourg
- Republic of Macedonia
- Madagascar
- Malawi
- Malaysia
- Maldives
- Mali
- Malta
- Martinique
- Mauritania
- Mauritius
- Mayotte
- Mexico
- Moldova, Republic of
- Monaco
- Mongolia
- Montenegro
- Montserrat
- Morocco
- Mozambique
- Namibia
- Nepal
- Netherlands
- New Zealand
- Nicaragua
- Niger
- Nigeria
- North Korea (Democratic Republic of Korea)
- Norway
- Oman
- Pacific Islands
- Pakistan
- Panama
- Papua New Guinea
- Paraguay
- Peru
- Philippines
- Poland
- Portugal
- Puerto Rico
- Qatar

- Reunion
- Romania
- Russian Federation
- Rwanda
- Saint Kitts and Nevis
- Saint Lucia
- Saint Vincent's & Grenadines
- Samoa
- Sao Tome and Principe
- Saudi Arabia
- Senegal
- Serbia
- Seychelles
- Sierra Leone
- Singapore
- Slovak Republic (Slovakia)
- Slovenia
- Solomon Islands
- Somalia
- South Africa
- South Korea (Republic of Korea)
- South Sudan
- Spain
- Sri Lanka
- Sudan
- Suriname
- Swaziland
- Sweden
- Switzerland
- Syria
- Tajikistan
- Tanzania
- Thailand
- Timor Leste
- Togo
- Trinidad & Tobago
- Tunisia
- Turkey
- Turkmenistan
- Turks & Caicos Islands
- Uganda
- Ukraine
- United Arab Emirates
- United States of America (USA)
- Uruguay
- Uzbekistan
- Venezuela
- Vietnam
- Virgin Islands (UK)
- Virgin Islands (US)
- Yemen
- Zambia
- Zimbabwe

If you responded ‘AUSTRALIA’ to Q20, please go to Q21.
All other responses, please go to Q20.1

Q20.1 In **what year did you arrive in Australia** to live for one year or more?

____

Q21 Have you attended **face-to-face training or education on refugee health** in the past 3 years?

- ◯ Yes
- ◯ No

Q21.1 If Yes, Can you provide some information about this? (e.g. when was the training, who organised it)

____

Q22 Have you participated in **on-line training or education on refugee health** in the past 3 years?

- ◯ Yes
- ◯ No

Q22.1 If Yes, Can you provide some information about this? (e.g. when was the training, who organised it)

____

Q23 If you have any other information that you think is important to capture, please include it here.

____

____

End of Individual Clinical Survey

(Post intervention/ 6 mo follow up)

Post intervention and 6-month follow up GP, Nurse and other clinical staff survey

Thank you for taking the time to complete this brief survey.

SECTION A: ASSESSMENT AND MANAGEMENT OF PATIENTS WITH REFUGEE BACKGROUNDS

Q1 Please rate your <u>confidence to undertake the following activities</u> with your patients with refugee backgrounds.

	Not at all	Slightly confident	Somewhat confident	Moderately confident	Very confident	Not my responsibility
Establish whether a patient is from a refugee background	○	○	○	○	○	○
Undertake a Refugee Health Assessment	○	○	○	○	○	○
Arrange an interpreter (on phone or in person)	○	○	○	○	○	○
Provide or support direct clinical care with an interpreter present (by phone or in person)	○	○	○	○	○	○

Q1.1 What <u>barriers</u>, if any, are there for your <u>use of interpreters</u>? Select all that apply.

- ☐ Cost
- ☐ Patient refusal
- ☐ Availability
- ☐ Patient concern about confidentiality
- ☐ No systems in place
- ☐ Issues with quality/training of interpreters
- ☐ Inability to get same sex interpreter
- ☐ Other, please specify ____

Q2 In the past 6 months, how often did you have <u>difficulty finding other services or providers to refer your patients with refugee backgrounds</u> for the following (if applicable)?

	Always or almost always	Often	Sometime s	Rarely or never	Don't know	Not applicable
Social and settlement issues	○	○	○	○	○	○
Mental health problems including PTSD	○	○	○	○	○	○
Oral and dental health issues	○	○	○	○	○	○

Q3 Do you use <u>HealthPathways or Map of Medicine for refugee care</u>?

○ Always or almost always ○ Often ○ Sometimes ○ Rarely or never ○ Don't know

Q4 Do you use <u>online resources</u> such as those provided by the Victorian Refugee Health Network or NSW Refugee Health Service <u>for refugee care</u>?

○ Yes ○ No ○ Don’t know ○ Not applicable

Q5 Please indicate the **extent to which you agree or disagree** with the following statements

	Strongly disagree	Disagree	Neither agree nor disagree	Agree	Strongly agree	No opinion or don't know
I find it difficult to provide care to patients who speak a different language to my own	○	○	○	○	○	○
Using professional interpreter services interferes with delivery of healthcare	○	○	○	○	○	○

SECTION B: FEEDBACK ON THE OPTIMISE PROJECT

Q6 Are you aware of your practice's involvement in the OPTIMISE Project?
The OPTIMISE Project is focused on improving the way in which the practices organises and delivers care to patients from refugee background

○ Yes ○ No ○ Don’t know

The OPTIMISE Project focused on four key areas: refugee identification, interpreter use, conduct of comprehensive health assessments and referral. Your practice has been making changes to clinical and non-clinical routines relating to each of these areas over the last 6 months.

Q7 Were you part of the team at your practice that was responsible for planning and implementing changes?

○ Yes, Go to Question 8 ○ No, Go to Question 9 ○ Don’t know

Q8 To what extent do you agree with the following statements

	Strongly disagree	Disagree	Somewhat disagree	Neither agree nor disagree	Somewhat agree	Agree	Strongly agree
The changes proposed as part of OPTIMISE Project action plans were easy to incorporate into the existing practice and clinical routines	○	○	○	○	○	○	○
The facilitator helped the practice to implement the action plans	○	○	○	○	○	○	○
The practice will be able to sustain the changes that were introduced as part of the OPTIMISE Project after the study ends	○	○	○	○	○	○	○
I would recommend the OPTIMISE Project to other practices	○	○	○	○	○	○	○

Q8.1 Please add any comments to your responses above here

____

____

____

____

____

Q9 To what extent do you agree with the following statements

	Strongly disagree	Disagree	Somewhat disagree	Neither agree nor disagree	Somewhat agree	Agree	Strongly agree
The changes proposed as part of the OPTIMISE Project action plans were easy to incorporate into the existing practice and clinical routines	○	○	○	○	○	○	○

Q9.1 Please add any comments to your responses above here

____

SECTION C: ABOUT YOU

Q10 What is your **name**?

____

Q11 Have you attended any **training or education on refugee health** in the past 6 months?
(Other than your involvement in the OPTIMISE project)

○ Yes ○ No

If yes,
Q11.1 Can you provide some information about this? (e.g. when was the training, who organised it)

____

Q12 If you have any other information that you think is important to capture, please include it here.

____

____

____
