# Supplementary material for: The OPTIMISE project: protocol for a mixed methods, pragmatic, quasi-experimental trial to improve primary care delivery to refugees in Australia
Source: BMC Health Serv Res. 2019 Jun 19;19:396. doi: 10.1186/s12913-019-4235-6 (PMC6585095; doi:10.1186/s12913-019-4235-6)
Supplement: Supplementary file 4 — Baseline Refugee Healthcare Survey (PDF 475 kb) [file 12913_2019_4235_MOESM4_ESM.pdf]

Baseline Refugee Healthcare Survey

Version date: 11h September 2017

Q1 **Name and address** of practice

____

____

____

Q2 Please indicate **who in the practice was involved in completing the survey**

____

____

____

____

Q3 Please note the names of **research team members involved in completing the survey**

____

SECTION A: NEW PATIENT PRESENTATIONS TO THE PRACTICE

Q4 How do **new patients with refugee backgrounds** currently **come to your practice**? (tick all that apply)

	Always or almost always	Often	Sometimes	Rarely or never	Don't know
Referred by settlement services	○	○	○	○	○
Referred by refugee health nurse	○	○	○	○	○
Referred by sponsor	○	○	○	○	○
Make their own appointment (or made by family or friends	○	○	○	○	○
Walk in	○	○	○	○	○

Q5 How does the practice **collect/confirm refugee or asylum seeker status** for a new patient? (e.g., from referral information; patients are routinely asked; If they are asked, when are they asked, who asks them, and where in the practice?)

____

____

____

Q6 Does the practice have any **written policies and protocols** about **recognising and recording patients with a refugee background**? If yes, please ask the respondents to provide copies after the survey has been completed.

- ○ Yes
- ○ No
- ○ Don't know

Q7 Does the practice **collect/confirm refugee patients' year of arrival** to Australia?

- ○ Yes
- ○ No
- ○ Don't know

Ask This Question:

If the response to Q7 is Yes

Q7.1 How does the practice **collect/confirm** their **year of arrival?** (i.e. how are patients asked – by whom, when, where?)

Q8 Are **new patients** asked what their **language** preferences are?

- ◯ Always or almost always
- ◯ Often
- ◯ Sometimes
- ◯ Rarely or never
- ◯ Don't know

Ask This Question:

If response to Q5 above is: Always or almost always OR Often OR Sometimes

Q8.1 **How are patients asked** about their language preferences?

(include details of who is responsible, when and where patients are asked about language preferences, and whether **all refugee patients** are asked)

____

____

____

Q9 Are **language preferences recorded** in a patient's medical record?

- ◯ Always or almost always
- ◯ Often
- ◯ Sometimes
- ◯ Rarely or never
- ◯ Don't know

Q10 Is the **need for an interpreter** recorded in a patient's medical record?

- ◯ Always or almost always
- ◯ Often
- ◯ Sometimes
- ◯ Rarely or never
- ◯ Don't know

Ask This Question:

If response to Q7 is: Always or almost always OR Often OR Sometimes

Q10.1 **How is the need for an interpreter recorded**? (include details of who is responsible, where in the medical record the information is noted, and whether the information is recorded for **refugee patients** in particular)

___

___

___

Q11 Does your practice **receive assistance from outside organisations** (like Primary Health Networks, Refugee Health Programs within the Local Health Authority) with **managing refugee patients**?

◯ Yes

◯ No

◯ Don't know

Ask This Question:

If response to Q8 is Yes

Q11.1 If yes, what organisations or services (please note regional differences)

☐ Refugee Health Fellow program

☐ Refugee Health Nurse program

☐ Local Primary Health Network (SEMPHN, NWMPHN or SWSPHN)

☐ Local Settlement Services (AMES Australia, Settlement Services International). If a different settlement service, please specify

____

☐ Victorian Refugee Health Network (Foundation House, Victorian Foundation for Survivors of Torture)

☐ Other, please specify

____

Q12 Research officer: *Record any comments about initial contact with patients with a refugee background made while answering the questions above or subsequently.*

____

____

SECTION B: USE OF INTERPRETING SERVICES

Q13 Does your practice use **professional translating and interpreting services** (TIS National)?

	Yes	No	Don't know	No need
By telephone	○	○	○	○
Face-to-face	○	○	○	○

Q14 In your opinion, are **GPs** in the practice generally **uncomfortable working with interpreters**?

- ○ Yes
- ○ No
- ○ Don't know

Q15 Has **training** on how to work with interpreters been offered to **GPs** by the practice?

- ○ Yes
- ○ No
- ○ Don't know

Ask This Question:

If response to Q13 is Yes

Q15.1 **Please provide details** about any training GPs may have received on working with interpreters.

e.g. what type of training, when & who provided it? How many GPs participated?

____

____

____

Q16 In your opinion, are **nurses and other clinical staff** in the practice generally **uncomfortable working with interpreters**?

◯ Yes

◯ No

◯ Don't know

Q17 Has **training** in how to work with interpreters been offered to **nurses and other clinical staff** by the practice?

◯ Yes

◯ No

◯ Don't know

Display This Question:

If the response to Q15 is Yes

Q17.1 **Please provide details** about any training nurses and other clinical staff may have received on working with interpreters.

e.g. what type of training, when & who provided it? How many nurses and other clinical staff participated?

____

____

____

Q18 In your opinion, are **reception staff** generally **uncomfortable working with interpreters**?

- Yes
- No
- Don't know

Q19 Has **training** on how to work with interpreters been offered to **reception staff** by the practice?

- Yes
- No
- Don't know

Display This Question:

If the response to Q17 is Yes

Q19.1 **Please provide details** about any reception staff may have received on working with interpreters. What type of training, when & who provided it? How many reception staff participated?

____

____

____

____

Q20 Does your **practice display and/or provide information to patients about the availability of interpreter services** at the practice? If yes, please ask the respondents to provide copies after the survey has been completed.

- ◯ Yes
- ◯ No
- ◯ Don't know

Q21 Does each clinician/office have a **speaker phone**?

- ◯ Yes
- ◯ No
- ◯ Don't know

Q22 Do phones have a **TIS sticker** on them?

- ◯ Yes
- ◯ No
- ◯ Don't know

Q23 Research officer: *Record any comments about use of interpreting services made while answering the questions above or subsequently.*

SECTION C: ASSESSMENTS AND HEALTHCARE

Q24 These questions relate to refugee health assessments

	Yes	No	Don't know	Not done in this practice
Does your practice use a Refugee Health Assessment template for screening people that have arrived in the last 12 months?	○	○	○	○
Does your practice set aside specific appointment times for completion of refugee health assessments?	○	○	○	○
Are longer appointments booked for refugee health assessments?	○	○	○	○
Is the practice nurse involved in refugee health assessments?	○	○	○	○

Q25 Research officer: *Record any comments about undertaking refugee health assessments made while answering the questions above or subsequently.*

____

____

____

SECTION D: REFERRAL PATHWAYS FROM THE PRACTICE FOR REFUGEE CLIENTS

Q26 Does your practice have a **documented process for referral of refugee patients to external services** i.e. specialists, support services, maternal and child health, dental etc. If yes,

please ask practice staff to provide copies of the documented process after the survey has been completed.

- ◯ Yes
- ◯ No
- ◯ Don't know

Q27 Do **staff help refugee patients (who need it) with referrals**, such as making an appointment or advising about potential costs.

- ◯ Always or almost always
- ◯ Often
- ◯ Sometimes
- ◯ Rarely or never
- ◯ Don't know

Q28 Does the practice have a **documented process to confirm whether refugee patients attended the services to which they were referred**?

If yes, please ask practice staff to provide copies of the documented process after the survey has been completed.

- ◯ Yes
- ◯ No
- ◯ Don't know

Q29 Does the practice have a **list of specialists and services that bulk bill** for refugee patient referrals?

- ◯ Yes
- ◯ No
- ◯ Don't know

Q30 Does the practice have a **list of specialists who are bilingual and what languages they speak**?

- ◯ Yes
- ◯ No
- ◯ Don't know

Ask This Question:

If the response to Q28 is Yes

Q30.1 How is the list of bilingual specialists and the languages they speak updated? How do GPs access the list?

____

____

Q31 Research officer: *Record any comments about referral pathways from the practice made while answering the questions above or subsequently.*

____

____

____

____

SECTION E: COMMUNICATION

Q32 Does your practice **adjust care to suit refugee patients with low literacy**? e.g. explain repeat scripts or use teach back?

- ◯ Yes
- ◯ No
- ◯ Don't know

Ask This Question:

If the response to Q30 is Yes

Q32.1 **Please give some examples** of how your practice adjusts care for refugee patients with low literacy

Q33 Do reception staff offer to **help refugee patients** when they are **filling out forms** required to support healthcare delivery (e.g. patient registration) ?

◯ Yes

◯ No

◯ Don't know

Q34 Is health and health service **information provided to refugee patients in simple English**, if required?

◯ Yes

◯ No

◯ Don't know

Q35 Is health and health service **information provided to refugee patients in languages other than English,** if required?

◯ Yes

◯ No

◯ Don't know

Q36 Does your practice **provide refugee patients with updated medication summaries** that describe in easy to understand language what medicines the patient is to take and how? If yes, please ask the respondents to provide an example of one of these lists after the survey has been completed.

◯ Yes

◯ No

◯ Don't know

Q37 Research officer: *Record any comments about communication with patients with refugee backgrounds made while answering the questions above or subsequently.*

___

___

___

SECTION F: EDUCATION AND TRAINING

Q38 Have practice staff received any **cultural competency training**?

- ◯ Yes
- ◯ No
- ◯ Don't know

Ask This Question:

If the response to Q36 is Yes

Q38.1 **Please give details** of any cultural competency training that practice staff may have received.

e.g. what type of training, when & who provided it? How many GPs, nurses and other staff participated?

___

___

___

___

Q39 Has the practice offered or supported access to any **education or training about refugee health to staff** over the past 12 months?

- ◯ Yes
- ◯ No
- ◯ Don't know

Ask This Question

If the response to Q37 is Yes

Q39.1 **Please give details** of refugee health training that practice staff may have received.

e.g. what type of training, when & who provided it? How many GPs, nurses and other staff participated?

____

____

____

____

Q40 Please describe any **other specific strategies that your practice uses when providing healthcare to patients with refugee (or refugee-like) backgrounds** here.

____

____

____

____

SECTION G: FINAL QUESTIONS

Q41 What does your practice **hope to get out of being involved** in the OPTIMISE project?

____

____

____

____

Q42 At the end of the project **how will you know that it was successful for your practice**? What changes in your practice would you like to see as a result of the project?

____

____

____

SECTION H: DOCUMENT CHECKLIST

Q43 Please check that the practice has provided copies of the following documents (if applicable)

- ☐ Policy/protocol for recognising refugee patients and recording this information
- ☐ Signage or patient handouts on the availability of interpreter services (flyers, information booklets, posters etc)
- ☐ Process for referral of refugee patients to external services
- ☐ Process for confirming refugee patients' attendance at services to which they are referred
- ☐ Example medication summary
- ☐ Other, please specify

____

End of Refugee Healthcare Survey

(Post intervention/6 mo follow up) OPTIMISE: Refugee Healthcare Survey

Instructions for researcher: Please review the practice's baseline refugee health care survey responses and the action plans to become familiar with the activities that the practice planned to undertake at the beginning of the intervention. Please take a printed copies of these documents with you to the visit.
When completing sections A-D, first check whether the practice made any changes to routines. If yes, ask all questions in the section as stated. If no, refer to the practice's responses to the questions at baseline at confirm that these responses are the same at post intervention and at 6-months follow up.

Q1 Name and address of practice

____

____

____

Q2 Please note the names and roles of practice staff involved in completing the survey

____

____

____

Q3 Please note the names of research team members involved in completing the survey

____

Q4 Have there been any changes to practice staff in the last 6 months?

○ Yes ○ No ○ Don't know

If yes, please explain in terms of discipline, gender, languages spoken and sessions worked. E.g. one new female, Dari speaking GP works at the practice three afternoons a week.

____

____

The questions in this survey relate to your practice's involvement in the OPTIMISE Project.

As part of this project, several members of your practice worked as a team with a refugee health expert to improve the ways in which your practice organises and delivers care to patients from refugee backgrounds.

SECTION A: REFUGEE PATIENTS AT YOUR PRACTICE

Q5 Has your practice changed the way in which you collect/confirm and record refugee or asylum seeker status for patients since taking part in the OPTIMISE Project?

- ◯ Yes, proceed to ask Q6 - Q12
- ◯ No, confirm practice responses to Q6, Q7 and Q12 are the same as at baseline.

Q6 Does the practice have any written policies and protocols about recognising and recording patients with a refugee background?

If yes, please ask the respondents to provide copies after the survey has been completed.

◯ Yes ◯ No ◯ Don't know

NEW PATIENTS

Q7 Does the practice collect/confirm refugee or asylum seeker status for a new patient?

◯ Always or almost always ◯ Often ◯ Sometimes ◯ Rarely or never ◯ Don't know

If response is always/almost always, often or sometimes
Q7.1 How is refugee or asylum seeker status for a new patient determined? (select all that apply)

- ☐ New patients as asked to state their country of birth or ethnicity and year of arrival in the new patient registration form
- ☐ New patients are asked whether they are from a refugee background in the new patient registration form (e.g. tick box)
- ☐ Staff ask new patients whether they are from a refugee background directly
- ☐ Other, please specify ____

EXISTING PATIENTS

Q8 Does the practice collect/confirm refugee or asylum seeker status for existing patients (if this information is missing)?

- ◯ Always or almost always
- ◯ Often
- ◯ Sometimes
- ◯ Rarely or never
- ◯ Don't know

If response is always/almost always, often or sometimes

Q8.1 How is refugee or asylum seeker status determined for existing patients? (select all that apply)

- ☐ Existing patients are given a form to fill which asks for country of birth or ethnicity and year of arrival
- ☐ Existing patients are given a form to fill which asks whether they are from a refugee background
- ☐ Staff ask existing patients whether they are from a refugee background directly
- ☐ Other, please specify ____

BOTH NEW AND EXISTING PATIENTS

Q9 Who is responsible for collecting information about refugee or asylum seeker status of both new and existing patients? (select all that apply)

- ◯ General practitioners
- ◯ Practice nurses
- ◯ Reception or administrative staff
- ◯ Other, please specify ________________________

Q10 Where is the information collected about refugee or asylum seeker status recorded? (select all that apply)

- ☐ Warnings box in Medical Director
- ☐ Reason for presentation field in Medical Director
- ☐ Country of birth and year of arrival fields in Pracsoft
- ☐ On screen comments box in Best Practice
- ☐ Ethnicity field in Best Practice
- ☐ Other social history box in Best Practice
- ☐ Diagnosis in Medical Director or Best Practice
- ☐ A separate refugee patient register (paper based or electronic)
- ☐ Other, please specify ______________________________________

Q11 Who is responsible for recording information about refugee or asylum seeker status status (select all that apply) Note: different staff members may be responsible for collecting and recording information

- ◯ General practitioners
- ◯ Practice nurses
- ◯ Reception or administrative staff
- ◯ Other, please specify ________________

Q12 Do the following staff have access to the information the practice has recorded about refugee or asylum seeker status?

	Yes, usually	Yes, sometimes	No	Don't know or not applicable
Reception or administrative staff	◯	◯	◯	◯
Practice nurses	◯	◯	◯	◯
Doctors	◯	◯	◯	◯
Other, please specify	◯	◯	◯	◯

Q13 Research officer: *Record any comments about identifying and recording whether patients are from a refugee background made while answering the questions above or subsequently.*

____

____

____

SECTION B: USE OF INTERPRETING SERVICES

Questions in this section relate to the practice's use of the Translating and Interpreting Service (TIS National) and/or informal interpreters such as bi-lingual staff and family members to communicate with patients with have limited English language proficiency, particularly those from refugee backgrounds.

Q14 Has your practice changed the way in which you work with refugees who have limited English language proficiency since taking part in the OPTIMISE Project?

- ○ Yes, proceed to ask Q15 - Q21
- ○ No, confirm practice responses to Q15, Q15.1, Q16, Q17, Q18, Q18.A.2, Q19 and Q20 are the same as at baseline.

Q15 Are new patients asked what their language preferences are?

○ Always or almost always ○ Often ○ Sometimes ○ Rarely or never ○ Don't know

If response is always/almost always, often or sometimes
Q15.1 Are language preferences recorded in a patient's medical record?

○ Always or almost always ○ Often ○ Sometimes ○ Rarely or never ○ Don't know

Q16 Does the practice confirm whether a patient needs an interpreter?

○ Always or almost always ○ Often ○ Sometimes ○ Rarely or never ○ Don't know

Q16.1 How does the practice confirm a patient's need for an interpreter? (select all that apply)

- ☐ Patients state their need for an interpreter when filling out the new patient registration form
- ☐ Patients show practice staff a TIS sticker or card which states that the patient's preferred language and their need for an interpreter
- ☐ Patients let practice staff know their preferred language by pointing it out on a TIS language card or poster displayed at the practice
- ☐ Family members and friends advise practice staff of the patient's language preferences and need for an interpreter
- ☐ Case worker or client liaison officer from a referring agency (e.g. settlement) advises practice staff of the patient's language preferences and need for an interpreter
- ☐ Other, please specify ____

Q16.2 Who is responsible for confirming whether a patient needs an interpreter?

- ○ General practitioners
- ○ Practice nurses
- ○ Reception or administrative staff
- ○ Other, please specify __________________________

Q16.3 Is the need for an interpreter recorded in a patient's medical record?

- ○ Always or almost always
- ○ Often
- ○ Sometimes
- ○ Rarely or never
- ○ Don't know

Q17 Does your practice display and/or provide information to patients about the availability of interpreter services at the practice?

If yes, please ask the respondents to provide copies after the survey has been completed.

- ○ Yes
- ○ No
- ○ Don't know

Q18 Does your practice use professional translating and interpreting services (TIS National)?

	Yes	No	Don't know	No need
By telephone	○	○	○	○
Face-to-face	○	○	○	○

If the practice does use TIS National by telephone or face to face

Q18.A.1 How does the practice book professional translating and interpreting services (TIS National)? (select all that apply)

- ☐ Reception or administrative staff pre-book interpreters via TIS Online
- ☐ Reception or administrative staff pre-book interpreters over the phone
- ☐ Reception or administrative staff contact interpreters over the phone just before the consultation starts
- ☐ General practitioner contacts interpreters over the phone during the consultation

Q18.A.2 Are practice nurses able to access professional translating and interpreting services (TIS National) for patients when needed?

○ Always or almost always ○ Often ○ Sometimes ○ Rarely or never ○ Don't know

If the practice does not use professional translating and interpreting services (TIS National)

Q18.B.1 How do staff communicate with patients from refugee backgrounds with limited English language proficiency? (select all that apply)

- ☐ General practitioners are bi-lingual/multi-lingual in one or more refugee community languages (1)
- ☐ Other staff at the practice are bi-lingual/multi-lingual in one or more refugee community languages (2)
- ☐ Patient's family members or friends translate for the patient (3)
- ☐ Other, please specify (4) ____

Q19 Does each clinician/office have a speaker phone?

○ Yes ○ No ○ Don't know

Q20 Do phones have a TIS sticker on them?

○ Yes ○ No ○ Don't know

Q21 Research officer: *Record any comments about use of interpreting services made while answering the questions above or subsequently.*

____

____

SECTION C: ASSESSMENTS AND HEALTHCARE

Q22 Has your practice changed the way in which you provide care to refugees since taking part in the OPTIMISE Project?

○ Yes, proceed to ask Q23 - 24

○ No, confirm practice responses to Q23 - 24 are the same as at baseline.

Q23 These questions relate to refugee health assessments

	Yes	No	Don't know	Not done in this practice
Does your practice use a Refugee Health Assessment template for screening people that have arrived in the last 12 months?	○	○	○	○
Does your practice set aside specific appointment times for completion of refugee health assessments?	○	○	○	○
Are longer appointments booked for refugee health assessments?	○	○	○	○
Is the practice nurse involved in refugee health assessments?	○	○	○	○

Q24 Does the practice use the Health Assessment Medicare items when providing care to refugee patients? For example, the MBS Item Numbers 701, 703, 705, 707 (noting that these are not specific to refugees)

○ Yes ○ No ○ Don't know

Q25 Research officer: *Record any comments about undertaking refugee health assessments made while answering the questions above or subsequently.*

____

____

____

____

SECTION D: REFERRAL PATHWAYS FROM THE PRACTICE FOR REFUGEE CLIENTS

Q26 Has your practice changed the way in which you refer refugees to external services since taking part in the OPTIMISE Project?

○ Yes, proceed to ask Q27 - 29

○ No, confirm practice responses to Q27 - 29 are the same as at baseline.

Q27 Do staff help refugee patients (who need it) with referrals, such as making an appointment or advising about potential costs.

○ Always or almost always ○ Often ○ Sometimes ○ Rarely or never ○ Don't know

Q28 Does the practice have a list of specialists and services that bulk bill for refugee patient referrals?

○ Yes ○ No ○ Don't know

Q29 Does the practice have a list of specialists who are bilingual and what languages they speak?

○ Yes ○ No ○ Don't know

If yes,
Q29.1 How is the list of bilingual specialists and the languages they speak updated? How do GPs access the list?

____

____

____

Q30 Research officer: *Record any comments about referral pathways from the practice made while answering the questions above or subsequently.*

____

____

____

____

____

SECTION E: EDUCATION AND TRAINING

Q31 Has the practice offered or supported access to any education or training about refugee health to staff over the past 6 months?

○ Yes ○ No ○ Don't know

If yes,

Q31.1 Please give details of refugee health training that practice staff may have received.
e.g. what type of training, when & who provided it? How many GPs, nurses and other staff participated?

____

____

____

SECTION F: FEEDBACK ON THE OPTIMISE PROJECT ACTIVITIES

Q32 What did the practice gain from being involved in the OPTIMISE project?

____

____

____

____

Q33 Please tell us to what extent you found the following aspects of the OPTIMISE project helped the practice to achieve what you set out to achieve

	Not at all helpful	Slightly helpful	Moderately helpful	Very helpful	Extremely helpful (essential to success)	Dont know or no opinion	This wasnt part of our experience
The face to face facilitation visits from a refugee health expert to help plan and make changes; provide support and resources.	○	○	○	○	○	○	○
The follow up phone contacts from a refugee health expert in between face to face visits to help troubleshoot issues arising	○	○	○	○	○	○	○
The availability of the facilitator beyond the planned facilitation visits and phone calls.	○	○	○	○	○	○	○
The action plan approach to planning change within the practice and monitoring progress	○	○	○	○	○	○	○
The resource book (printed or electronic) with information on how to evidence based refugee health care	○	○	○	○	○	○	○
The cheat sheet with links to key resources on evidence based refugee health care	○	○	○	○	○	○	○
The PENCS CAT4 Refugee Health data which showed what the practice was doing well and what the practice could improve upon at the start of the project	○	○	○	○	○	○	○

Q34 Overall, to what extent did the OPTIMISE project change the ways in which your practice:

	Not at all	A little	Quite a lot	A great deal
identifies patients from refugee backgrounds and records this information in the patients' medical records	○	○	○	○
identifies refugee patients' who need an interpreter and organise professional interpreting services for these patients	○	○	○	○
conducts comprehensive physical and mental health assessments using the Refugee Health Assessment template	○	○	○	○
identifies and refers refugee patients to appropriate health and social welfare services in the region	○	○	○	○

Q35 Please provide an additional comments/feedback in the space below

____

____

____

SECTION G: DOCUMENT CHECKLIST

Q36 Please check that the practice has provided copies of the following documents (if applicable)

- ☐ Policy/protocol for recognising refugee patients and recording this information
- ☐ Signage or patient handouts on the availability of interpreter services (flyers, information booklets, posters etc)
- ☐ Process for referral of refugee patients to external services
- ☐ Process for confirming refugee patients' attendance at services to which they are referred
- ☐ Other, please specify ____
